# Treatment seeking and antibiotic use for urinary tract infection symptoms in the time of COVID-19 in Tanzania and Uganda

**DOI:** 10.7189/jogh.14.05007

**Published:** 2024-01-19

**Authors:** Emmanuel Olamijuwon, Katherine Keenan, Martha F Mushi, Catherine Kansiime, Eveline T Konje, Mike Kesby, Stella Neema, Benon Asiimwe, Stephen E Mshana, Kathryn J Fredricks, Benjamin Sunday, Joel Bazira, Alison Sandeman, Derek J Sloan, Joseph R Mwanga, Wilber Sabiiti, Matthew TG Holden, CARE Consortium

**Affiliations:** 1 School of Geography and Sustainable Development, University of St Andrews, St Andrews, UK; 2 Catholic University of Health and Allied Sciences, Mwanza, Tanzania; 3 School of Public Health, College of Health Sciences, Makerere University Kampala, Uganda; 4 Department of Sociology and Anthropology, Makerere University, Kampala, Uganda; 5 Department of Medical Microbiology, Makerere University College of Health Sciences, Kampala, Uganda; 6 Department of Microbiology, Mbarara University of Science and Technology, Mbarara, Uganda; 7 School of Medicine, University of St Andrews, St Andrews, UK

## Abstract

**Background:**

There is still little empirical evidence on how the outbreak of coronavirus disease 2019 (COVID-19) and associated regulations may have disrupted care-seeking for non-COVID-19 conditions or affected antibiotic behaviours in low- and middle-income countries (LMICs). We aimed to investigate the differences in treatment-seeking behaviours and antibiotic use for urinary tract infection (UTI)-like symptoms before and during the pandemic at recruitment sites in two East African countries with different COVID-19 control policies: Mbarara, Uganda and Mwanza, Tanzania.

**Methods:**

In this repeated cross-sectional study, we used data from outpatients (pregnant adolescents aged >14 and adults aged >18) with UTI-like symptoms who visited health facilities in Mwanza, Tanzania and Mbarara, Uganda. We assessed the prevalence of self-reported behaviours (delays in care-seeking, providers visited, antibiotics taken) at three different time points, labelled as ‘pre-COVID-19 phase’ (February 2019 to February 2020), ‘COVID-19 phase 1’ (March 2020 to April 2020), and ‘COVID-19 phase 2’ (July 2021 to February 2022).

**Results:**

In both study sites, delays in care-seeking were less common during the pandemic than they were in the pre-COVID phase. Patients in Mwanza, Tanzania had shorter care-seeking pathways during the pandemic compared to before it, but this difference was not observed in Mbarara, Uganda. Health centres were the dominant sources of antibiotics in both settings. Over time, reported antibiotic use for UTI-like symptoms became more common in both settings. During the COVID-19 phases, there was a significant increase in self-reported use of antibiotics like metronidazole (<30% in the pre-COVID-19 phase to 40% in COVID phase 2) and doxycycline (30% in the pre-COVID-19 phase to 55% in COVID phase 2) that were not recommended for treating UTI-like symptoms in the National Treatment Guidelines in Mbarara, Uganda.

**Conclusions:**

There was no clear evidence that patients with UTI-like symptoms attending health care facilities had longer or more complex treatment pathways despite strict government-led interventions related to COVID-19. However, antibiotic use increased over time, including some antibiotics not recommended for treating UTI, which has implications for future antimicrobial resistance.

Antimicrobial resistance (AMR) is a global public health issue which makes infections more costly to treat and common health care interventions such as surgeries potentially life-threatening [1–3]. The burden of drug-resistant infections is estimated to be highest in low- and middle-income regions, particularly Africa [4], where infectious disease accounts for a higher share of morbidity and mortality, and where health care infrastructures are under-resourced compared with high-income regions. It is estimated that by 2050, AMR could cause low-income countries to lose more than 5% of their gross domestic product (GDP) and push millions into poverty [5]. AMR in low- and middle-income countries (LMICs) is driven by multiple factors, ranging from inappropriate use of antibiotics (including over-prescription, over-use of antibiotics and non-adherence to clinical advice [6]) and unprescribed purchase thereof [7], a lack of affordable clinical diagnostics [8,9], environmental factors such as sanitation [10,11], as well as socio-economic drivers like health care costs for patients [12].

Researchers have debated whether the coronavirus disease 2019 (COVID-19) pandemic intersects with the ongoing ‘silent pandemic’ of AMR, especially in LMICs [13–15]. Some suggested that travel restrictions, school, workplace, and non-essential service closures, improved hygiene practices, and enhanced public health information dissemination during the pandemic could decrease infection rates [16], reduce antibiotics use, and slow AMR [15]. In several high-income countries, COVID-19-related restrictions were associated with a substantial reduction in community dispensing of antibiotics primarily used to treat respiratory infections [17–21]. However, disruptions to health care access, financial hardships associated with pandemic restrictions, the fear of catching COVID-19, and the associated stigma may have motivated individuals to seek care outside the formal health care facilities or to self-medicate with antibiotics [13,14,22,23]. In Australia, for example, COVID-19-induced psychological distress was positively related to antibiotic self-medication [24]. In Iran, the fear of catching COVID-19 resulted in increased postponement of dental visits and antibiotic self-medication compared to pre-pandemic periods [25]. In sub-Saharan Africa, attempts to understand the indirect impacts of the pandemic have focused on service delivery, covering areas such as human immunodeficiency virus (HIV), chronic care, maternal, neonatal, and child health [26–33], but with limited evidence on patients’ experiences of care seeking for infections that often require antibiotics. Existing studies have offered mixed findings depending on context and the type of health care service. For example, antenatal and child health care seeking was delayed and reduced in Uganda, and there were periodic shortages of amoxicillin [26]. Conversely, services such as postnatal and HIV care experienced no disruptions in services or reductions in patient numbers [27,34,35].

In this study, we aimed to investigate changes in patient treatment-seeking for urinary tract infection (UTI) during the outbreak of COVID-19 at two sites in East Africa: Mbarara, Uganda and Mwanza, Tanzania. Understanding treatment pathways and antibiotic use in these countries is particularly important because drug sellers commonly sell them for these illnesses without prescription [7,36]. Despite commonalities in health care structures in these neighbouring countries, there were marked differences in government-led COVID-19 related interventions and policies (**Figure 1**), thus making a valuable comparative case study. The Ugandan government implemented strict household lockdowns and restricted travel and activities for key periods post-2020, while restrictions in Tanzania have been less consistent. Based on other studies from the region [26,30], we anticipated that formal health care use would be delayed and reduced during COVID-19 periods. We also anticipated that government-led COVID-19 prevention efforts would contribute to an increase in self-treatment, use of drug sellers, and related inappropriate antibiotics use, compared with pre-COVID-19. Given that strict lockdowns were more common in Uganda, we anticipated more obvious effects on UTI treatment-seeking there than in Tanzania.

**Figure 1:**
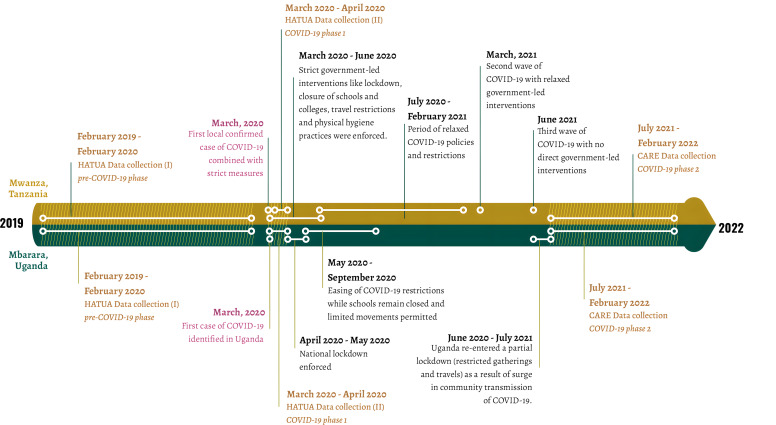
Timeline of recruitment and COVID-19 restrictions at the two study sites. The shaded area and brown-coloured texts refer to the period of data collection at both study sites.

## METHODS

### Data

In this repeated cross-sectional study, we used data collected by the Holistic Approach to Unravelling Antibacterial Resistance (HATUA) and COVID-19 and Antibacterial Resistance in East-Africa (CARE) consortia, which use UTI – a commonly occurring bacterial infection – as a clinical prism to investigate the drivers of antibacterial resistance [37]. The consortia recruited patients presenting with UTI-like symptoms at health facilities (including government and private/faith-based-run health facilities) in Kenya, Tanzania, and Uganda. The facilities ranged from community health centres (levels 2–3) to regional referral/zonal referral hospitals (levels 4/5). In this study, we used a subset of data collected at health facilities in Mwanza, Tanzania, and Mbarara, Uganda over three time points between January 2019 and February 2022 and labeled these as ‘pre-COVID-19 phase’ (February 2019 to February 2020), ‘COVID-19 phase 1’ (March 2020 to April 2020), and ‘COVID-19 phase 2’ (July 2021 to February 2022) (**Figure 1**). Our data collection periods also coincided with the pre-COVID-19 period, the early months of the pandemic, and 15–22 months later. Using standardised recruitment protocols and tools, doctors identified patients with symptomatic UTI for inclusion in the study. Patients answered a questionnaire on treatment-seeking for UTI-like symptoms, antibiotic use practices, and socio-demographic characteristics. Our analysis sample comprised 3666 individuals recruited in three waves (2408 in Mwanza, Tanzania and 1258 in Mbarara, Uganda).

### Variables

#### Socio-demographic characteristics

Age was categorised into young adults (14–34 years), middle-aged adults (35–54 years), and older adults (≥55 years). We coded sex as male/female and highest educational attainment into ‘no formal education,’ ‘primary,’ ‘secondary,’ and ‘tertiary.’

#### Treatment seeking

All patients responded to a structured questionnaire (detailed elsewhere [38]) on their treatment-seeking behaviours for their recent UTI-like symptoms. We first asked them how long it took them to seek care after the onset of their UTI-like symptoms and coded individuals as having ‘delayed treatment’ if they waited for two weeks or more and ‘no delay’ if two weeks or less. The patients provided details on the treatment steps before their current visit to the health facility, including the type of provider (government clinic, private clinic, drug shop or pharmacy, self-treated at home) and type of medication taken. Patients reported medications using a drug card/drug bag method [39], which we used to create a binary response variable indicating whether antibiotics had been taken before coming to the recruitment health facility.

### Statistical analysis

We used descriptive statistics to summarise the patients’ sociodemographic characteristics, treatment behaviours, and antibiotics most commonly taken before the current health care visit. We used chi-squared (χ^2^) tests of homogeneity to compare their demographic characteristics and behaviour patterns across the different study phases and sites. We used chord diagrams to visualise more detailed flows of patient treatment seeking. Chord diagrams are a visualisation method used in migration studies [40–44] and population health research [45,46] that represents connections between several nodes as curved arcs within a circle. Each node in the chord diagram is a treatment attempt with a certain type of provider (that is, government clinic, private clinic, drug shops/pharmacies, self-treatment) and accounts for whether antibiotics were taken. We produced diagrams using the ‘circlize’ package in R [47]. We also fitted multivariable logistic regression models to assess significant differences in antibiotic use behaviour while adjusting for sociodemographic characteristics, including age, sex, education, and investigated variations by interacting country/time period. From the regression coefficients, we generated predicted probabilities and compared these across the three periods and countries. Finally, we compared patterns and trends in the use of specific antibiotics across the periods and countries, paying particular attention to antibiotics recommended for use in National Treatment Guidelines for UTI [48,49]. All statistical analyses were done in R, version 4.2.1. (R Core Team, Vienna, Austria) within the RStudio environment, version 2022.12.0 + 353 (Posit, PBC, Boston, Massachusetts, USA) [50,51]. We considered *P* < 0.05 as statistically significant.

## RESULTS

### Socio-demographic profile of patients

The socio-demographic characteristics of patients varied across the study periods in both sites (**Table 1**). About 13% of the patients recruited during the pre-COVID-19 phase in Mwanza, Tanzania were aged >55, compared to about 23% and 22% of patients recruited in COVID-19 phases 1 and 2, respectively. In the same study site, the proportion of patients with both the lowest and highest educational attainment nearly doubled in the COVID-19 phase 1, compared to the pre-COVID-19 period; however, in COVID-19 phase 2, educational distribution had returned to levels similar to pre-COVID-19 patterns. We observed a similar pattern in Mbarara, Uganda: there were proportionally more highly educated patients in COVID-19 phase 1, but this returned to pre-COVID-19 levels in phase 2. In Mwanza, Tanzania, there were proportionally more males in the patient samples recruited during the pandemic than in the pre-pandemic period.

**Table 1:** Descriptive statistics on patients recruited from both sites – presented as percentages

	Mwanza, Tanzania				Mbarara, Uganda			
	**Pre-COVID-19 phase (n = 1295)**	**COVID-19 phase 1 (n = 231)**	**COVID-19 phase 2 (n = 882)**	***P*-value†**	**Pre-COVID-19 phase (n = 506)**	**COVID-19 phase 1 (n = 170)**	**COVID-19 phase 2 (n = 582)**	***P*-value†**
**Age**				<0.001				0.009
Young adults (18-34)	58.4	42.0	48.1		53.2	64.7	51.4	
Middle-aged adults (35-54)	28.6	34.6	30.4		37.9	30.0	36.6	
Older adults (55+)	13.1	23.4	21.5		8.9	5.3	12.0	
**Gender**				<0.001				0.210
Male	17.3	32.0	27.8		20.0	25.3	23.7	
Female	82.7	68.0	72.2		80.0	74.7	76.3	
**Highest educational attainment**				<0.001				<0.001
No formal education	10.3	19.9	7.82		14.2	14.7	8.93	
Primary	57.7	44.6	61.2		57.1	47.1	65.1	
Secondary	25.9	24.2	24.9		21.5	22.9	19.9	
Tertiary	6.10	11.3	6.01		7.11	15.3	6.0	
**Delayed seeking treatment**				<0.001				<0.001
No delay/less than two weeks delay	67.2	73.2	87.2		51.6	67.6	82.0	
Delay of more than two weeks	32.8	26.8	12.8		48.4	32.4	18.0	
**First thing tried**				<0.001				<0.001
Came to this clinic today	35.4	38.5	67.7		26.1	35.9	28.5	
Government clinic	39.6	30.7	20.4		33.2	33.5	38.5	
Private clinic	12.8	12.6	5.1		27.7	18.8	27.8	
Drug shop or pharmacy	8.3	10.4	3.3		5.5	3.5	1.7	
Self-treatment	3.9	7.8	3.5		7.5	8.3	3.4	
Taken antibiotics anytime in the pathway*				<0.001				<0.001
No	58.1	40.8	46.0		41.7	31.2	19.2	
Yes	41.9	59.2	54.0		58.3	68.8	80.8	

*Sample excludes patients who came straight to the health facility to treat the reported symptoms.

†χ^2^ tests of homogeneity used to examine whether the demographic characteristics and behaviour patterns of patients follow the distribution across the different study phases.

### Treatment-seeking pathways

We also noted differences in the treatment behaviours of patients recruited across the study periods (**Table 1**). During the pre-COVID-19 period, about 33% of patients in Mwanza, Tanzania and 48% of patients in Mbarara, Uganda had delays of two weeks or more, while fewer patients had delays during COVID-19 phase 2 (13% Mbarara, Uganda and 18% Mwanza, Tanzania). In Mwanza, Tanzania, the proportion of patients who came directly to the clinic for their symptoms increased across the study phases, while the proportion of patients who had taken antibiotics prior to presenting at a clinic increased over the study periods in both study sites.

**Figure 2:**
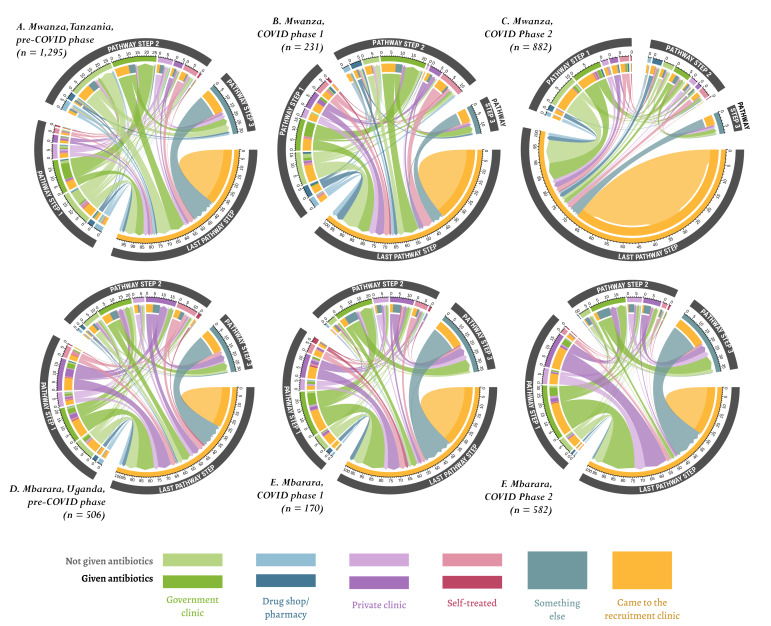
Treatment-seeking pathways during COVID-19. The chords represent percentages of the total sample, so the wider the circle’s segments, the higher the percentage of patients taking those steps. The outer gray ring distinguishes the different steps (steps 1–3, and last step). The second ring shows the relative share of patients who accessed treatment from that provider overall. Finally, the innermost coloured bars depict the share of patients moving from their current step to another one.

We visualised the sequence of steps taken according to types of providers and types of medicine across sites and recruitment periods in the chord diagram (**Figure 2** and Figure S1 in the **Online Supplementary Document**) and the related percentages separately (Table S1-S2 in the **Online Supplementary Document**). Considering Mwanza, Tanzania only (**Figure 2**, Panels A-C), we found that treatment-seeking complexity declined over the three periods, evidenced by a declining percentage of patients who had taken multiple steps to treat their UTI symptoms and an increase in those coming straight to the clinic. We did not observe this trend in Mbarara, Uganda. Regardless of the site or period, most respondents went to government and private health centres as their first point of care. However, over time, there were differences in the share of patients choosing clinics over self-care. In Mwanza, Tanzania, the proportion of patients who self-treated at home doubled (from 4%-8%) between the pre-COVID-19 and COVID-19 phase 1 period, but declined to 4% by COVID-19 phase 2. In Mbarara, Uganda, however, the proportion of patients who had self-treated at home during COVID-19 phase 2 declined significantly to 3% from 8% during the pre-COVID-19 and COVID-19 phases.

### Antibiotic use patterns before and during COVID-19

**Figure 3:**
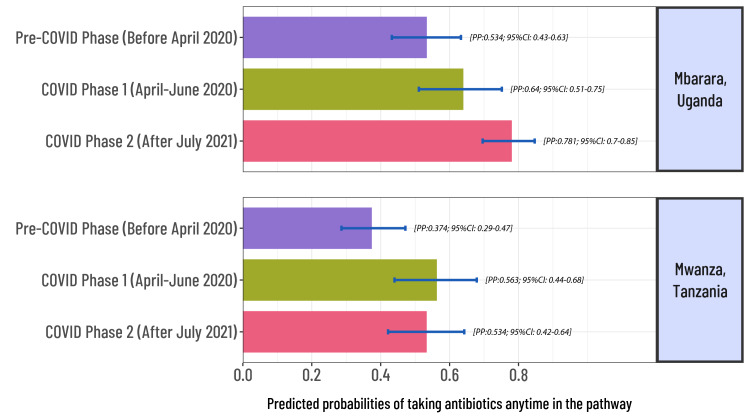
Predicted probabilities of taking ABs anytime in the pathway among patients who tried treating their UTI symptoms before coming to the recruitment clinic. PP – predicted probabilities, CI – confidence interval.

**Figure 4:**
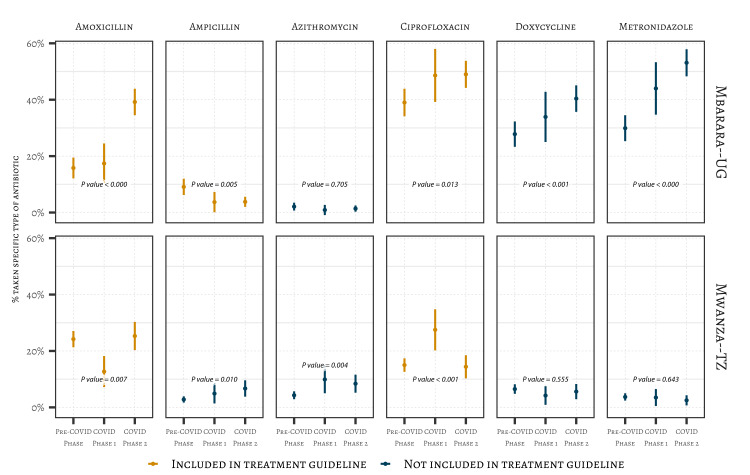
Distribution of patients by the type of antibiotics they took to treat their UTI symptoms. Mwanza, TZ: pre-COVID-19 phase (n = 836); Mwanza, TZ: COVID-19 phase 1 (n = 142); Mwanza, TZ: COVID-19 phase 2 (n = 285); Mbarara, UG: pre-COVID-19 phase (n = 374); Mbarara, UG: COVID-19 phase 1 (n = 109); Mbarara, UG, COVID-19 phase 2 (n = 416). *P*-values are derived from χ^2^ tests.

After adjusting for the socio-demographic characteristics at both sites, patients recruited in the COVID-19 phases were more likely to have taken antibiotics for UTI-like symptoms prior to recruitment to the study (**Figure 3**). In Mbarara, Uganda, the adjusted predicted probability of taking antibiotics anytime in the pathway increased (53% pre-COVID-19 phase, 64% COVID-19 phase 1 and 78% COVID-19 phase 2). Similar increases were observed in Mwanza, Tanzania (38% pre-COVID-19 phase, 58% COVID-19 phase 1 and 56% during COVID-19 phase 2). Over time, there was a significant increase in the use of antibiotics not recommended for treating UTIs in national treatment guidelines. In Mbarara, Uganda, for example, metronidazole use increased from 30% to 53% and doxycycline from 28% to 40% between the pre-COVID-19 phase and COVID-19 phase 2. In Mwanza, Tanzania, the use of ampicillin increased from 3% to 7% and azithromycin from 4% to 8% between the pre-COVID-19 phase and COVID-19 phase 1 (**Figure 4**).

## DISCUSSION

In this study, we investigated changes in treatment-seeking behaviour for UTI-like symptoms between pre-COVID-19 and the pandemic period in Uganda and Tanzania. We observed that patients who visited the recruitment clinics during the pandemic were less likely to have delayed treatment seeking and overall took fewer treatment steps, particularly in Mwanza, Tanzania compared to Mbarara, Uganda. Reduced delays in seeking care during COVID-19 could have resulted from patients fearing going to other community providers like drug sellers, wanting to take more direct routes to care, or having fewer barriers than usual to seeking timely care at facilities. The finding is not completely consistent across outcomes and time periods. For example, we observed marginal increases in reported rates of self-treatment and use of drug sellers in Mwanza, Tanzania but not Mbarara, Uganda during the COVID-19 phase 1. However, the overall trend of treatment pathways becoming less complex over the pandemic periods is somewhat contrary to our expectations. This could be explained in several ways. Taking the data at face value, it could be that UTI care was one of the less disrupted services. While there were documented interruptions in antenatal care and sexual health services in Uganda in 2020 [26], other services such as postnatal care [26], maternal and child health services [52,53] were seemingly not interrupted, while studies from other parts of sub-Saharan Africa document stability in health care provision and consistent patient attendance [30,32,54]. Additionally, our findings could also have been affected by selection bias. By using data from health care facility attendees, we likely underestimate the extent of self-medication and use of other providers because self-mediators whose symptoms resolve easily do not appear in our sample.

Although this simplification of patient care-seeking might predict more successful treatment outcomes, the increase in overall antibiotic use during the COVID-19 periods at both sites suggests otherwise. As a very prevalent disease commonly treated with antibiotics, data on UTI might be suggestive of community-level antibiotic use and drug resistance. Given the common misuse of antibiotics highlighted previously [7], the observed increase in antibiotic use among UTI patients is a cause for concern and may be suggestive of increasing AMR.

We also observed a significant increase in the proportion of patients treating UTI symptoms with antibiotics, such as metronidazole and doxycycline, that were not recommended for treating UTI in either country [48,49,55]. This increase was more obvious in Mbarara, Uganda, which experienced stricter government-led restrictions than Mwanza, Tanzania, and where antibiotics are commonly sold on demand [36]. Further investigation is necessary to determine if these changes are related to the pandemic or represent more generalised shifts in the prevalence of UTI in the population or medication use over time. Whatever the underlying drivers, this has important implications for AMR, as it has been well established that longer duration and multiple courses of antibiotic treatment further drive AMR [56].

The study has some limitations. First, our COVID-19 data collection periods did not coincide exactly with severe lockdowns (**Figure 1**), so patients in our study may have felt fewer concerns about attending health care facilities and not faced serious barriers. Our data collection periods in UG and TZ were slightly different, which could inhibit comparability. Moreover, the COVID-19 phase 1 data collection period covered a few months, while the comparable data collection periods were longer. Although continuous data collection could have solved these issues, it was not practical due to resource constraints and COVID-19 restrictions. Treatment-seeking data were self-reported and could be subject to response bias, and confusion on the part of patients about symptoms and medicines. Finally, our study design was observational and thus did not allow any causal inference for us to attribute behavioural patterns to the COVID-19 restrictions.

## CONCLUSIONS

Treatment pathways for UTI care were less complex during the pandemic compared to the pre-pandemic period. However, we found evidence of increased antibiotic use, especially of those antibiotics not recommended for the treatment of UTI symptoms. Given what is known about the sub-optimal dispensing and use of antibiotics, both could further accelerate AMR. Our results emphasise the need to further investigate factors driving antibiotic use and AMR in these settings and to ensure antibiotic stewardship is resilient to future disease outbreaks.

## Additional material


Online Supplementary Document
