# Supplementary material for: Treatment seeking and antibiotic use for urinary tract infection symptoms in the time of COVID-19 in Tanzania and Uganda
Source: J Glob Health. 2024 Jan 19;14:05007. doi: 10.7189/jogh.14.05007 (PMC10795859; doi:10.7189/jogh.14.05007)
Supplement: Online Supplementary Document [file jogh-14-05007-s001.pdf]

Treatment seeking and antibiotic use for urinary tract infection symptoms in the time of COVID-19 in Tanzania and Uganda

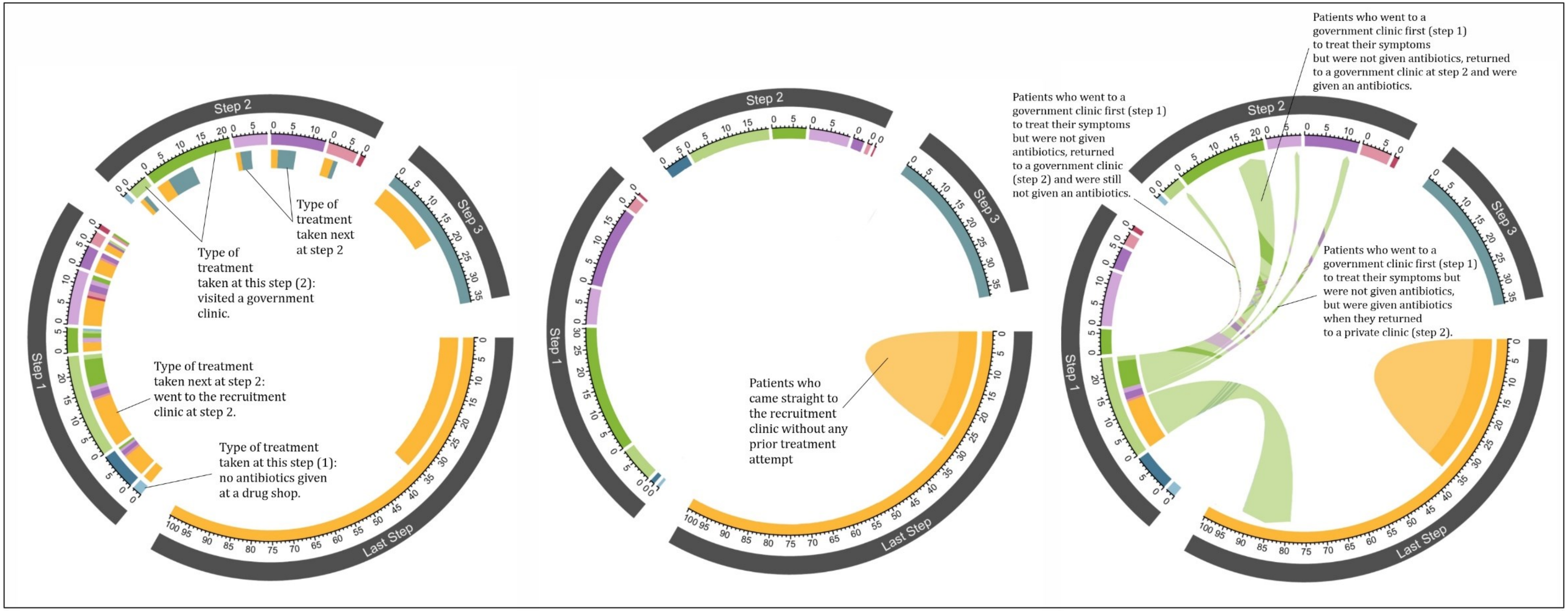

Figure S1: Supplementary Table for Chord Diagram, Uganda

Table S1: Supplementary Table for Chord Diagram, Uganda

Origin	Destination	Pre-COVID-19Phase	COVID-19Phase 1	COVID-19Phase 2
Step0 Came to the recruitment clinic	Step0 Came to the recruitment clinic	26.1	35.9	28.5
Step1 Drug Shop/Pharmacy_fal	Step0 Came to the recruitment clinic	2.0	0.0	0.5
Step1 Drug Shop/Pharmacy_fal	Step2 Drug Shop/Pharmacy_fal	0.2	0.0	0.0
Step1 Drug Shop/Pharmacy_fal	Step2 Government Clinics_fal	0.4	0.0	0.0
Step1 Drug Shop/Pharmacy_fal	Step2 Private Clinics_fal	0.2	0.0	0.0
Step1 Drug Shop/Pharmacy_fal	Step2 Private Clinics_tru	0.2	0.0	0.0
Step1 Drug Shop/Pharmacy_tru	Step0 Came to the recruitment clinic	2.2	2.4	0.7
Step1 Drug Shop/Pharmacy_tru	Step2 Drug Shop/Pharmacy_tru	0.2	0.0	0.0
Step1 Drug Shop/Pharmacy_tru	Step2 Private Clinics_tru	0.2	0.0	0.0
Step1 Government Clinics_fal	Step0 Came to the recruitment clinic	7.3	5.9	5.5
Step1 Government Clinics_fal	Step2 Government Clinics_fal	1.0	0.0	0.9
Step1 Government Clinics_fal	Step2 Government Clinics_tru	2.2	0.6	0.3
Step1 Government Clinics_fal	Step2 Private Clinics_fal	1.0	0.6	0.9
Step1 Government Clinics_fal	Step2 Self-Treated_fal	0.8	0.6	0.2
Step1 Government Clinics_tru	Step0 Came to the recruitment clinic	11.3	12.9	19.6
Step1 Government Clinics_tru	Step2 Drug Shop/Pharmacy_fal	0.2	0.0	0.0
Step1 Government Clinics_tru	Step2 Drug Shop/Pharmacy_tru	0.2	0.0	0.0
Step1 Government Clinics_tru	Step2 Government Clinics_fal	1.2	1.2	1.2
Step1 Government Clinics_tru	Step2 Government Clinics_tru	3.2	7.1	5.0
Step1 Government Clinics_tru	Step2 Private Clinics_fal	0.6	1.2	1.4
Step1 Government Clinics_tru	Step2 Private Clinics_tru	2.8	1.8	2.2
Step1 Government Clinics_tru	Step2 Self-Treated_fal	1.2	0.6	0.5
Step1 Government Clinics_tru	Step2 Self-Treated_tru	0.4	0.0	0.3
Step1 Private Clinics_fal	Step0 Came to the recruitment clinic	3.0	2.4	6.4
Step1 Private Clinics_fal	Step2 Drug Shop/Pharmacy_fal	0.4	0.6	0.0
Step1 Private Clinics_fal	Step2 Government Clinics_fal	1.0	0.6	0.3
Step1 Private Clinics_fal	Step2 Government Clinics_tru	1.2	1.2	0.7
Step1 Private Clinics_fal	Step2 Private Clinics_fal	0.8	1.2	0.5
Step1 Private Clinics_fal	Step2 Private Clinics_tru	1.0	0.0	0.7
Step1 Private Clinics_fal	Step2 Self-Treated_fal	1.0	0.0	0.0
Step1 Private Clinics_fal	Step2 Self-Treated_tru	0.2	0.0	0.0
Step1 Private Clinics_tru	Step0 Came to the recruitment clinic	8.1	7.1	13.1
Step1 Private Clinics_tru	Step2 Drug Shop/Pharmacy_tru	0.6	0.0	0.2
Step1 Private Clinics_tru	Step2 Government Clinics_fal	1.0	0.0	0.5
Step1 Private Clinics_tru	Step2 Government Clinics_tru	3.0	1.2	3.4
Step1 Private Clinics_tru	Step2 Private Clinics_fal	0.6	1.2	0.9
Step1 Private Clinics_tru	Step2 Private Clinics_tru	4.9	1.8	0.9
Step1 Private Clinics_tru	Step2 Self-Treated_fal	0.8	1.2	0.3
Step1 Private Clinics_tru	Step2 Self-Treated_tru	0.2	0.6	0.0
Step1 Self-Treated_fal	Step0 Came to the recruitment clinic	3.2	3.5	2.1
Step1 Self-Treated_fal	Step2 Government Clinics_fal	0.4	0.0	0.0
Step1 Self-Treated_fal	Step2 Government Clinics_tru	0.6	0.0	0.2
Step1 Self-Treated_fal	Step2 Private Clinics_fal	0.2	0.0	0.2
Step1 Self-Treated_fal	Step2 Self-Treated_fal	2.4	0.6	0.2
Step1 Self-Treated_fal	Step2 Self-Treated_tru	0.2	0.0	0.0
Step1 Self-Treated_tru	Step0 Came to the recruitment clinic	0.4	1.8	0.2
Step1 Self-Treated_tru	Step2 Government Clinics_tru	0.2	0.0	0.0
Step2 Drug Shop/Pharmacy_fal	Step0 Came to the recruitment clinic	0.6	0.6	0.0
Step2 Drug Shop/Pharmacy_fal	Step3 Something else	0.2	0.0	0.0
Step2 Drug Shop/Pharmacy_tru	Step0 Came to the recruitment clinic	0.6	0.0	0.0
Step2 Drug Shop/Pharmacy_tru	Step3 Something else	0.4	0.0	0.2
Step2 Government Clinics_fal	Step0 Came to the recruitment clinic	3.4	1.2	0.7
Step2 Government Clinics_fal	Step3 Something else	1.6	1.2	2.2
Step2 Government Clinics_tru	Step0 Came to the recruitment clinic	5.5	3.5	2.1
Step2 Government Clinics_tru	Step3 Something else	4.7	7.1	7.7
Step2 Private Clinics_fal	Step0 Came to the recruitment clinic	2.4	1.2	0.7
Step2 Private Clinics_fal	Step3 Something else	1.0	2.9	3.3
Step2 Private Clinics_tru	Step0 Came to the recruitment clinic	4.5	1.8	0.9
Step2 Private Clinics_tru	Step3 Something else	4.5	4.7	4.0
Step2 Self-Treated_fal	Step0 Came to the recruitment clinic	3.6	2.4	0.3
Step2 Self-Treated_fal	Step3 Something else	2.6	1.2	1.0
Step2 Self-Treated_tru	Step0 Came to the recruitment clinic	1.0	0.0	0.2
Step2 Self-Treated_tru	Step3 Something else	0.0	0.6	0.3
Step3 Something else	Step0 Came to the recruitment clinic	15.0	17.7	18.7

NOTE: tru means that an antibiotic was taken, while fal means that an antibiotic was not taken after consultation with the specific care provider

Table S2: Supplementary Table for Chord Diagram, Uganda

Origin	Destination	Pre-COVID Phase	COVID Phase 1	COVID Phase 2
Step0 Came to the recruitment clinic	Step0 Came to the recruitment clinic	35.4	38.5	67.7
Step1 Drug Shop/Pharmacy_fal	Step0 Came to the recruitment clinic	1.9	2.6	1.1
Step1 Drug Shop/Pharmacy_fal	Step2 Drug Shop/Pharmacy_fal	1.2	0.4	0.1
Step1 Drug Shop/Pharmacy_fal	Step2 Government Clinics_fal	1.0	0.4	0.0
Step1 Drug Shop/Pharmacy_fal	Step2 Government Clinics_tru	0.1	0.0	0.0
Step1 Drug Shop/Pharmacy_fal	Step2 Private Clinics_fal	0.3	0.0	0.0
Step1 Drug Shop/Pharmacy_fal	Step2 Private Clinics_tru	0.1	0.0	0.0
Step1 Drug Shop/Pharmacy_fal	Step2 Self-Treated_fal	0.4	1.3	0.1
Step1 Drug Shop/Pharmacy_tru	Step0 Came to the recruitment clinic	1.6	3.9	1.5
Step1 Drug Shop/Pharmacy_tru	Step2 Drug Shop/Pharmacy_fal	0.2	0.4	0.0
Step1 Drug Shop/Pharmacy_tru	Step2 Drug Shop/Pharmacy_tru	0.6	0.9	0.1
Step1 Drug Shop/Pharmacy_tru	Step2 Government Clinics_fal	0.2	0.0	0.0
Step1 Drug Shop/Pharmacy_tru	Step2 Government Clinics_tru	0.5	0.4	0.0
Step1 Drug Shop/Pharmacy_tru	Step2 Private Clinics_fal	0.2	0.0	0.1
Step1 Drug Shop/Pharmacy_tru	Step2 Self-Treated_fal	0.1	0.0	0.2
Step1 Government Clinics_fal	Step0 Came to the recruitment clinic	6.6	8.7	8.3
Step1 Government Clinics_fal	Step2 Drug Shop/Pharmacy_fal	0.8	0.0	0.1
Step1 Government Clinics_fal	Step2 Drug Shop/Pharmacy_tru	0.3	0.9	0.1
Step1 Government Clinics_fal	Step2 Government Clinics_fal	8.3	2.2	0.8
Step1 Government Clinics_fal	Step2 Government Clinics_tru	0.9	1.3	0.8
Step1 Government Clinics_fal	Step2 Private Clinics_fal	1.0	0.4	0.1
Step1 Government Clinics_fal	Step2 Private Clinics_tru	0.2	0.9	0.1
Step1 Government Clinics_fal	Step2 Self-Treated_fal	1.6	1.3	0.2
Step1 Government Clinics_fal	Step2 Self-Treated_tru	0.2	0.0	0.0
Step1 Government Clinics_tru	Step0 Came to the recruitment clinic	5.3	6.1	6.1
Step1 Government Clinics_tru	Step2 Drug Shop/Pharmacy_fal	0.2	0.4	0.1
Step1 Government Clinics_tru	Step2 Drug Shop/Pharmacy_tru	0.6	0.0	0.5
Step1 Government Clinics_tru	Step2 Government Clinics_fal	1.9	0.0	0.3
Step1 Government Clinics_tru	Step2 Government Clinics_tru	10.0	7.4	1.2
Step1 Government Clinics_tru	Step2 Private Clinics_fal	0.3	0.0	0.3
Step1 Government Clinics_tru	Step2 Private Clinics_tru	0.8	0.0	0.5
Step1 Government Clinics_tru	Step2 Self-Treated_fal	0.5	1.3	0.7
Step1 Government Clinics_tru	Step2 Self-Treated_tru	0.1	0.0	0.1
Step1 Private Clinics_fal	Step0 Came to the recruitment clinic	2.8	3.5	1.2
Step1 Private Clinics_fal	Step2 Drug Shop/Pharmacy_fal	0.6	0.0	0.0
Step1 Private Clinics_fal	Step2 Drug Shop/Pharmacy_tru	0.2	0.0	0.0
Step1 Private Clinics_fal	Step2 Government Clinics_fal	1.2	0.9	0.2
Step1 Private Clinics_fal	Step2 Government Clinics_tru	0.7	0.0	0.1
Step1 Private Clinics_fal	Step2 Private Clinics_fal	1.6	0.0	0.5
Step1 Private Clinics_fal	Step2 Private Clinics_tru	0.5	0.0	0.1
Step1 Private Clinics_fal	Step2 Self-Treated_fal	0.8	0.0	0.1
Step1 Private Clinics_tru	Step0 Came to the recruitment clinic	1.7	4.3	1.8
Step1 Private Clinics_tru	Step2 Drug Shop/Pharmacy_fal	0.1	0.0	0.1
Step1 Private Clinics_tru	Step2 Drug Shop/Pharmacy_tru	0.2	0.0	0.3
Step1 Private Clinics_tru	Step2 Government Clinics_fal	0.2	0.0	0.1
Step1 Private Clinics_tru	Step2 Government Clinics_tru	0.4	1.3	0.2
Step1 Private Clinics_tru	Step2 Private Clinics_fal	0.7	0.0	0.1
Step1 Private Clinics_tru	Step2 Private Clinics_tru	0.8	2.2	0.0
Step1 Private Clinics_tru	Step2 Self-Treated_fal	0.2	0.4	0.1
Step1 Private Clinics_tru	Step2 Self-Treated_tru	0.1	0.0	0.0
Step1 Self-Treated_fal	Step0 Came to the recruitment clinic	1.2	3.0	1.5
Step1 Self-Treated_fal	Step2 Drug Shop/Pharmacy_tru	0.1	0.9	0.5
Step1 Self-Treated_fal	Step2 Government Clinics_fal	0.5	0.4	0.3
Step1 Self-Treated_fal	Step2 Government Clinics_tru	0.1	0.4	0.5
Step1 Self-Treated_fal	Step2 Private Clinics_fal	0.1	0.0	0.0
Step1 Self-Treated_fal	Step2 Private Clinics_tru	0.2	0.0	0.0
Step1 Self-Treated_fal	Step2 Self-Treated_fal	0.8	1.3	0.2
Step1 Self-Treated_tru	Step0 Came to the recruitment clinic	0.2	0.9	0.3
Step1 Self-Treated_tru	Step2 Drug Shop/Pharmacy_tru	0.1	0.0	0.1
Step1 Self-Treated_tru	Step2 Government Clinics_fal	0.1	0.0	0.0
Step1 Self-Treated_tru	Step2 Government Clinics_tru	0.2	0.0	0.0
Step1 Self-Treated_tru	Step2 Private Clinics_fal	0.1	0.0	0.0
Step1 Self-Treated_tru	Step2 Self-Treated_tru	0.2	0.0	0.0
Step2 Drug Shop/Pharmacy_fal	Step0 Came to the recruitment clinic	1.9	1.7	0.6
Step2 Drug Shop/Pharmacy_fal	Step3 Something else	1.1	0.0	0.0
Step2 Drug Shop/Pharmacy_tru	Step0 Came to the recruitment clinic	1.0	1.7	1.5
Step2 Drug Shop/Pharmacy_tru	Step3 Something else	1.1	0.9	0.1
Step2 Government Clinics_fal	Step0 Came to the recruitment clinic	8.9	3.5	1.1
Step2 Government Clinics_fal	Step3 Something else	4.6	0.4	0.7
Step2 Government Clinics_tru	Step0 Came to the recruitment clinic	8.8	7.4	1.5
Step2 Government Clinics_tru	Step3 Something else	4.0	3.5	1.4
Step2 Private Clinics_fal	Step0 Came to the recruitment clinic	1.8	0.4	0.5
Step2 Private Clinics_fal	Step3 Something else	2.5	0.0	0.7
Step2 Private Clinics_tru	Step0 Came to the recruitment clinic	1.5	1.7	0.1
Step2 Private Clinics_tru	Step3 Something else	1.2	1.3	0.6
Step2 Self-Treated_fal	Step0 Came to the recruitment clinic	3.5	5.2	1.6
Step2 Self-Treated_fal	Step3 Something else	0.8	0.9	0.1
Step2 Self-Treated_tru	Step0 Came to the recruitment clinic	0.3	0.0	0.1
Step2 Self-Treated_tru	Step3 Something else	0.2	0.0	0.0
Step3 Something else	Step0 Came to the recruitment clinic	15.5	7.0	3.6

NOTE: tru means that an antibiotic was taken, while fal means that an antibiotic was not taken after consultation with the specific care provider

Table S3: Multivariate logistic regression model showing the association of complex treatment-seeking behaviour with socio-demographic and clinically relevant characteristics.

	Took antibiotics anytime			
	Uganda	Tanzania	Combined Model A	Combined Model B
Early COVID-19(April-December 2020)	1.58* (1.00-2.55)	2.22*** (1.53-3.23)	1.88*** (1.40-2.52)	
Late COVID-19(June 2021-February 2022)	2.94*** (2.12-4.09)	1.80*** (1.36-2.39)	2.24*** (1.82-2.77)	
Country: Uganda			2.25*** (1.86-2.73)	
Gender: Female	0.65** (0.44-0.96)	1.08 (0.82-1.41)	0.91 (0.73-1.14)	0.91 (0.73-1.13)
Age: 35-54 yrs	1.19 (0.85-1.67)	0.71** (0.55-0.93)	0.87 (0.71-1.07)	0.87 (0.71-1.07)
Age: 55+ yrs	0.58** (0.34-0.99)	0.60*** (0.43-0.85)	0.61*** (0.45-0.80)	0.60*** (0.45-0.80)
Education: Primary	1.75** (1.11-2.73)	1.53** (1.05-2.26)	1.62*** (1.22-2.17)	1.59*** (1.19-2.12)
Education: Secondary	1.82** (1.04-3.17)	2.02*** (1.33-3.11)	1.95*** (1.40-2.72)	1.93*** (1.39-2.70)
Education: Tertiary	1.28 (0.65-2.57)	2.23*** (1.32-3.79)	1.77*** (1.17-2.70)	1.78*** (1.17-2.71)
Early COVID-19(Apr-Jun, 2020): Tanzania				2.17*** (1.50-3.15)
Late COVID-19(Jun-Feb, 2022) : Tanzania				1.75*** (1.32-2.31)
Pre- COVID-19(< Apr, 2020) : Uganda				1.98*** (1.54-2.54)
Early COVID-19(Apr-Jun, 2020) : Uganda				3.02*** (1.97-4.71)
Late COVID-19(Jun-Feb, 2022) : Uganda				6.00*** (4.54-8.02)
Constant	1.25 (0.67-2.33)	0.51*** (0.31-0.83)	0.52*** (0.35-0.76)	0.55*** (0.37-0.81)
Observations	899	1,263	2,162	2,162
Log Likelihood	-514.57	-842.86	-1,370.13	-1,365.06
Akaike Inf. Crit.	1,047.14	1,703.71	2,760.27	2,754.12
Note:				*p**p***p<0.01
